# Patterns of regional lymph node metastasis of nasopharyngeal carcinoma: A meta-analysis of clinical evidence

**DOI:** 10.1186/1471-2407-12-98

**Published:** 2012-03-21

**Authors:** Francis CH Ho, Ivan WK Tham, Arul Earnest, Khai Mun Lee, Jiade J Lu

**Affiliations:** 1 Department of Radiation Oncology, National University Cancer Institute, National University Health System, 5 Lower Kent Ridge Road, Singapore 119074, Republic of Singapore; 2 Centre for Quantitative Medicine, Duke-NUS Graduate Medical School, 8 College Road, Singapore, Singapore 169857

**Keywords:** Nasopharyngeal cancer, Lymph nodes, Lymphatic metastasis, Meta-analysis

## Abstract

**Background:**

The characteristics of cervical lymphatic metastasis in nasopharyngeal carcinoma (NPC) are not completely understood. As such, radiotherapy to the entire lymphatic of the neck bilaterally has been empirically practiced even in early stage disease, although not supported by clinical evidence. We studied the pattern and probability of nodal metastasis through a meta-analysis of published evidences, with an aim to establish an evidence-based guideline for selecting and delineation of clinical target volume of neck lymphatics for conformation radiation for NPC.

**Methods:**

A literature search yielded an initial 411 original articles, and 13 studies with 2920 NPC cases staged via MRI were included in this analysis. The occurrence of nodal metastasis was calculated and analyzed according to the respective regional nodal levels.

**Results:**

85% of NPC cases presented with lymphadenopathy. The most commonly involved regions include retropharyngeal (69%) and level II lymph nodes (70%). The overall probability of levels III, IV, and V nodal involvement are 45%, 11%, and 27%, respectively. Low-risk node groups included the supraclavicular, levels IA/IB and VI nodes, and parotid nodes with involvement rates at 3%, 0%, 3%, 0%, and 1%, respectively. Nodal metastases followed an orderly pattern and the probability of "skip" metastasis between levels varied between 0.5-7.9%.

**Conclusions:**

Lymph node metastasis in NPC follows a predictable and orderly pattern. The rarity of metastasis in certain nodal groups and "skip" metastasis suggest that reduced treatment volume is feasible in conformal radiotherapy for NPC.

## Background

Nasopharyngeal carcinoma (NPC) is the most commonly diagnosed head and neck cancer in Southeast Asia, with a reported annual incidence of 30-80 per 10^5 ^population in endemic regions [1]. Like most other squamous cell carcinomas (SCC) of the head and neck primaries, lymphatic drainage of the nasopharynx is predominantly to the cervical lymph nodes. However, NPC has the highest preponderance for regional lymph node metastasis among head and neck SCC [2]. Radiation has been the mainstay of definitive treatment for NPC. The fields of radiation therapy for NPC traditionally encompass the primary disease and involved neck nodes, as well as the entire draining lymphatic regions to the lower neck. In a retrospective study reported by Lee *et al*, 57 (30%) of the 189 patients who did not receive elective neck irradiation subsequently developed cervical lymph node recurrence. However, none of the seven regionally treated patients relapsed [3]. Results from this and other similar studies have led to the practice of empirical irradiation of the entire neck in treating NPC,[4] regardless of the stage of NPC at diagnosis.

However, treatment of a large field to the neck is associated with substantial morbidities, both early and late: Early toxicities include brisk radiation dermatitis and odynophagia, especially if concurrent chemo-radiation is utilized; late toxicities may include neck fibrosis, lymphedema, brachial plexopathy, and thyroid dysfunction [5]. The therapeutic ratio may be maintained or improved if selective neck irradiation can be safely implemented in patients with limited nodal disease burden. Such practice may improve the tolerability of radiation therapy, as well as the compliance and quality of life of the patients.

A more accurate definition of target volume of regional lymph node region in radiation therapy also becomes possible because of the significant improvements made in imaging technology. Compared to computed tomography (CT), magnetic resonance imaging (MRI) has improved soft tissue contrast and multi-planar capability [6]. MRI scans have been shown to be particularly useful in the assessment of retropharyngeal and cervical lymphadenopathy [7]. Ng et al found that the nodal status was changed from negative on CT to positive on MRI in 4 of 67 patients (6%). This led them to conclude that MRI allows more accurate evaluation of the extent of NPC than CT and should be the primary mode of investigation [8]. Sakata et al also showed that MR was better than CT at identifying metastases to lymph nodes in the carotid and retropharyngeal spaces, with significant prognostic implications [9]. Liao et al demonstrated a significant difference between CT and MRI in demonstrating involvement in the retropharyngeal lymph nodes (CT, 52.1% vs. MRI, 69.0%). MRI resulted in changes in 10.7% of N stage cases and 38.6% of clinical stage cases [6]. A small study involving patients suspected of having NPC has demonstrated that MRI had a sensitivity of 100%, specificity of 95%, negative predictive value of 100%, positive predictive value of 43%, and an overall accuracy of 95% when verified with biopsy. (AJNR Am J Neuroradiol. 2006 Jun-Jul;27(6):1288-91. Magnetic resonance imaging for the detection of nasopharyngeal carcinoma. King AD, Vlantis AC, Tsang RK, Gary TM, Au AK, Chan CY, Kok SY, Kwok WT, Lui HK, Ahuja AT) On the basis that MRI has a high overall accuracy rate, possesses good imaging characteristics, and is the current standard of care, we choose to focus our efforts on MRI for this study.

Advances in radiation therapy, including image guidance and intensity-modulated radiation therapy (IMRT), have also allowed oncologists to be highly selective and accurate in treatment delivery. In the IMRT era, it is often up to the clinical judgment of the radiation oncologist to decide how much of the neck to irradiate and to what dose [10]. While a standardized atlas [11] is already in routine clinical use for the delineation clinical target volumes in the neck, there is currently no consensus as to the optimal volume for elective irradiation of the neck for NPC, especially for patients with node negative disease.

Several authors have described the pattern of nodal metastases in NPC,[2,12,13] with a common view that cervical node metastases appear to occur in an orderly fashion with infrequent skip metastases. However, the actual distribution of nodal metastases as described in terms of lymph node levels differs between studies. Additionally, the reported rate of "skip" metastases varies between studies, ranging from 0.5% to 7.9% [14,15]. As such, we embarked on this review to examine the pattern and probability of regional node metastasis through a systematic analysis of published evidence using MRI for diagnosis and staging of NPC. Additionally, we sought to identify low risk regional node groups in NPC, thereby providing an evidence-based proposal for lymphatic target selection in conformal radiation therapy for NPC.

## Methods

### Search strategy and eligibility criteria

A systematic review of original articles and abstracts analyzing the cervical nodal metastasis status of patients with NPC was performed by searching electronic databases PUBMED (January 1990 to December 2009), CANCERLIT (January 1990 to December 2009), and the Cochrane Library (January 1980 to July 2007). Studies were eligible if the cervical and/or retropharyngeal node positivity rate in NPC was reported. Search strategy included the following keywords in various combinations: "nasopharyngeal cancer", "lymph nodes", "nasopharyngeal carcinoma", "lymphatic metastasis", "cervical nodes", and "retropharyngeal nodes". Searches were supplemented by scanning bibliographies and references of included articles. The titles and abstracts of articles retrieved by this search were evaluated against inclusion criteria, and the manuscripts of all studies deemed potentially eligible were obtained.

The imaging modality used had to be predominantly or exclusively magnetic resonance imaging. Studies using CT only were excluded. There was no restriction criterion on the number of patients enrolled in the study. Given the volume of articles retrieved, articles were limited to English only. As there were overlapping and duplicate data sets detected on the same series of patients, only the most recent or most informative study was included in the analysis after checking with the respective authors.

### Data extraction

Two investigators, namely the first and the last author, independently extracted data from selected articles, including year of publication, first author, reported retropharyngeal and cervical LN positive rate in NPC patients at the respective nodal stations as and when available. To ensure the accuracy of this process and to minimize subjective judgment, all data were verified between the two reviewers, and discrepancies were settled through consensus discussion. Two participants of this analysis examined the accuracy of the data from each individual publication.

Multiple criteria were used to determine metastatic lymph node involvement, namely, central necrosis, extra capsular spread, shortest diameter of cervical or medial retropharyngeal lymph nodes > 1 cm and > 5 mm for lateral retropharyngeal lymph node(s). The occurrence of LN metastasis (retrospectively classified according to DAHANCA, EORTC, GORTEC, NCIC, RTOG consensus guidelines as far as possible) was calculated and analyzed according to the respective regional lymph nodal stations [11]. Studies where nodal stations could not be retrospectively classified were excluded from this meta-analysis. The incidence of disease involvement of all regional lymph nodal regions (according to RTOG classification) was the primary outcome. In this way, pooled analyses of the incidence of metastasis to regional lymph nodes were calculated and reported.

### Data analysis

Statistical analyses were performed using Stata^® ^software, version 10.2 (Stata Corp College Station, TX, USA). Publication bias for the primary endpoint was assessed via construction of a Begg's funnel plot, as well as by the Begg and Mazumdar adjusted rank correlation method [16]. Meta-analysis for the regional node metastasis in NPC was performed by calculating pooled estimates of proportion. Lymph node metastasis positive rate was the primary outcome. Using the Cochran Q Statistic, we assessed inter-study heterogeneity. This is calculated as the weighted sum of squared differences between individual study effects and the pooled effect across studies, with the weights being those used in the pooling method [17]. Because studies were found to be heterogeneous, proportion of patients with positive LN metastasis for the respective nodal station with corresponding 95% confidence intervals (CIs) were calculated using random-effects modeling after DerSimonian and Laird [18]. The CIs were calculated using formulae for proportion. Studies were weighted using random effects analysis. The weightage of each study was a combination of sample size (i.e. within study variation) and between study variations.

## Results

### Characteristics of included studies

The initial literature search resulted in 411 citations using the data extraction methods as described above. The title and abstract of each retrieved publication were reviewed to confirm that the article reported on the incidence of lymph node involvement in patients with NPC. In the event that this approach was not informative, the full article was retrieved and reviewed in detail. This process resulted in excluding 379 studies and 33 studies were selected. Of these 33 studies, the main modality of imaging was MRI in 19 studies, CT in 13 studies and PET in 2 studies. Out of the 19 articles focusing on MRI, four were non-English articles and were excluded. Two articles from Wang *et al *were found to have overlapping data after verifying with the original author; the more relevant paper of the two was chosen [19]. One article by Liao *et al *was excluded as the incidence of node metastasis was described as the total no of positive lymph nodes in the population as opposed to other papers, which described the number of patients with positive lymph nodes at the respective nodal stations [6].

**Figure 1:**
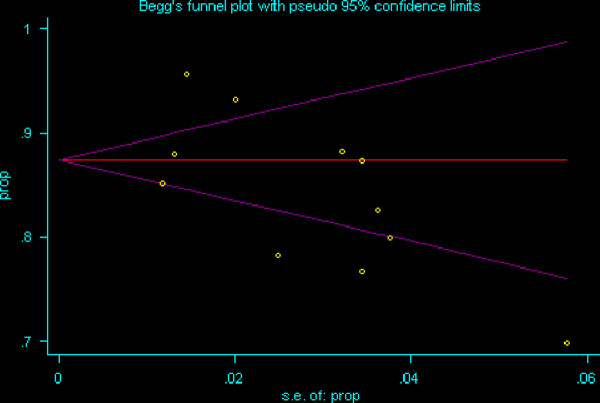
**Begg's Funnel Plot assessing publication bias for the proportion of patients presenting with any LN metastasis in 11 relevant studies**. Yellow dots represent the 11 relevant studies. Y axis is the proportion of patients in the particular study presenting with any lymph node metastasis. X axis is the standard error of this proportion of interest.

Figure 1 demonstrates the Begg's Funnel Plot assessing the publication bias for the proportion of patients with any nodal metastases in 11 studies. Only 11 studies out of the 13 studies were included in this plot, as the remaining two studies did not include relevant data for patients presenting with any lymphadenopathy. The funnel plot shows that a few points fall outside the funnel, but they are both above and below the funnel, hence indicating no clear direction in the bias, and a formal Egger test also indicates that there is no significant publication bias (p = 0.143).

Consequently, thirteen original research reports (as listed in Table 1 below) and a total of 2920 NPC cases evaluated with MRI were included in this analysis [7,8,12-14,19-26]. Three out of 13 studies were prospective studies, while the rest were retrospective studies that had looked at consecutive patients [12,23,25]. The patients were diagnosed with NPC between 1990 and 2006. The mean number of patients per study was 224 with a range of 63 patients to 924 patients.

**Table 1:** Characteristics of the 13 included studies

No	First Author	n	Any LN	Cervical LN	RLN	Level I LN	Level Ib LN	Level II LN	Level III LN	Level lV LN	Level V LN	Level VI LN	Parotid L	SCF LN
**1**	**Tang**	924	786		679		24	590	226	56	87		6	31
**2**	**Wang**	618	543	508	392	508	21	506	237	72	200		7	
**3**	**Liu**	275	215	175	175	175		174	65	16	18		4	8
**4**	**King**	150	115	115	108	115	3				66		2	
**5**	**Ng**	202	193		162			190	172	22				31
**6**	**Fuwa**	94	82	82		82								
**7**	**Ng**	101	89		73		2	85	54	31	24	2	3	20
**8**	**Lu**	159	148	139	108	139								
**9**	**Lam**	44			39			13						
**10**	**Chong**	114	91	58	59	58								
**11**	**Ng**	67		42	39	42								
**12**	**Kam**	63	44	44		44								
**13**	**Wolden**	109	90	90		90								

Abbreviations: LN, lymph nodes; RLN, Retropharyngal LN; SCF, supraclavicular fossa.

In studies that provided baseline demographic information on NPC patients, a total of 2101 were men and 702 were women [7,8,12-14,19-26]. The mean age was reported in 4 studies and ranged between 47.8 years and 49.3 years,[7,8,12,23] and the reported median age ranged from 45 years to 51 years in 6 studies [14,20-22,25,26]. In the seven studies that provided data on the histological subtypes of the patients, most patients had either WHO type II or III NPC. This ranged from 82% to 100%, with type III being more common [7,13,14,19-21,26]. Other histological subtypes like adenocarcinoma were rarely reported [14]. Of the six studies that looked at cervical LN metastasis specifically, all utilized all of the criteria for nodal involvement detailed above [7,12-14,19,20]. The LN location was classified either according to the consensus guidelines [11] or the "Level" system by Som *et al. *[27] The only major differences between these two guidelines lie in their classification of supraclavicular nodes. This may explain why the studies using the Som classification demonstrated a much higher rate of supraclavicular nodes involvement at more than 15%[12,20] compared to the studies that used the consensus guidelines, which reported rates of approximately 3% [13,14]. Hence the data for supraclavicular LN involvement in this meta-analysis has to be interpreted with caution.

### Results by lymph node levels

**Figure 2:**
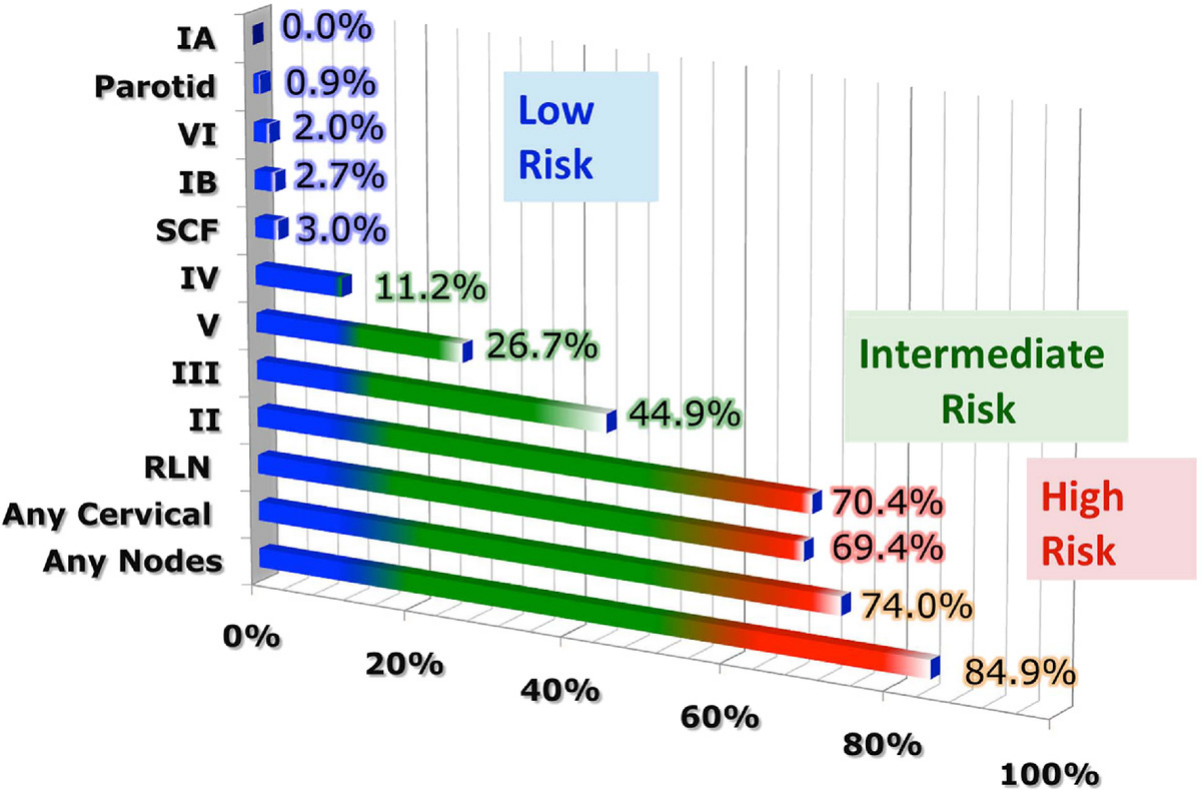
**Graphical representation of results divided into respective lymph node echelons and risk groups**. The X axis percentages represent the percentage of nodal involvement at the respective level.

**Figure 3:**
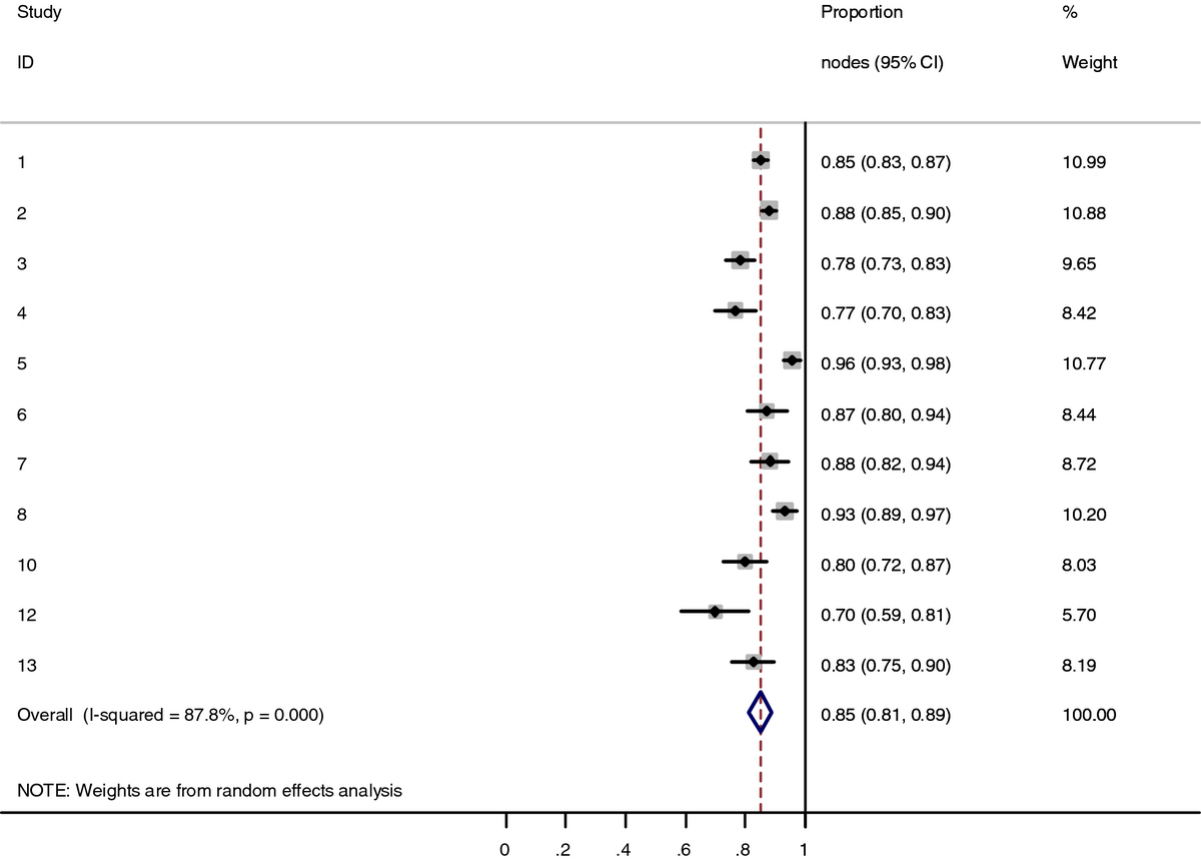
**Meta-analysis plot for patients with any LN metastasis**. This is the forest plot generated from the 11 studies that contained relevant data. Notice study 9 and 11 are missing as these 2 studies did not contain data specifying they looked at both retropharyngeal lymph nodes and cervical lymph nodes. The 1^st ^column specifies the study set used. The second column specified the proportion of patients that presented with lymph node metastasis in the particular study. The last column states the weightage of each study contributing to the meta-analysis. The X axis represents the proportion of patients who present with any nodal involvement.

**Figure 4:**
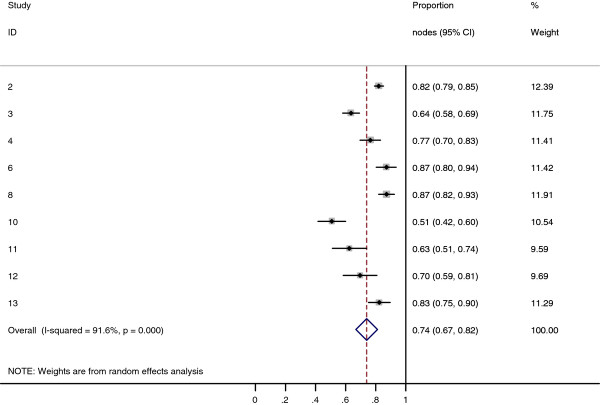
**Meta-analysis plot for patients with any cervical LN metastasis**. This is the forest plot generated from the 9 studies that contained relevant data. The 1^st ^column specifies the study set used. The second column specified the proportion of patients that presented with lymph node metastasis in the particular study. The last column states the weightage of each study contributing to the meta-analysis. The X axis represents the proportion of patients who present with any cervical LN involvement.

**Figure 5:**
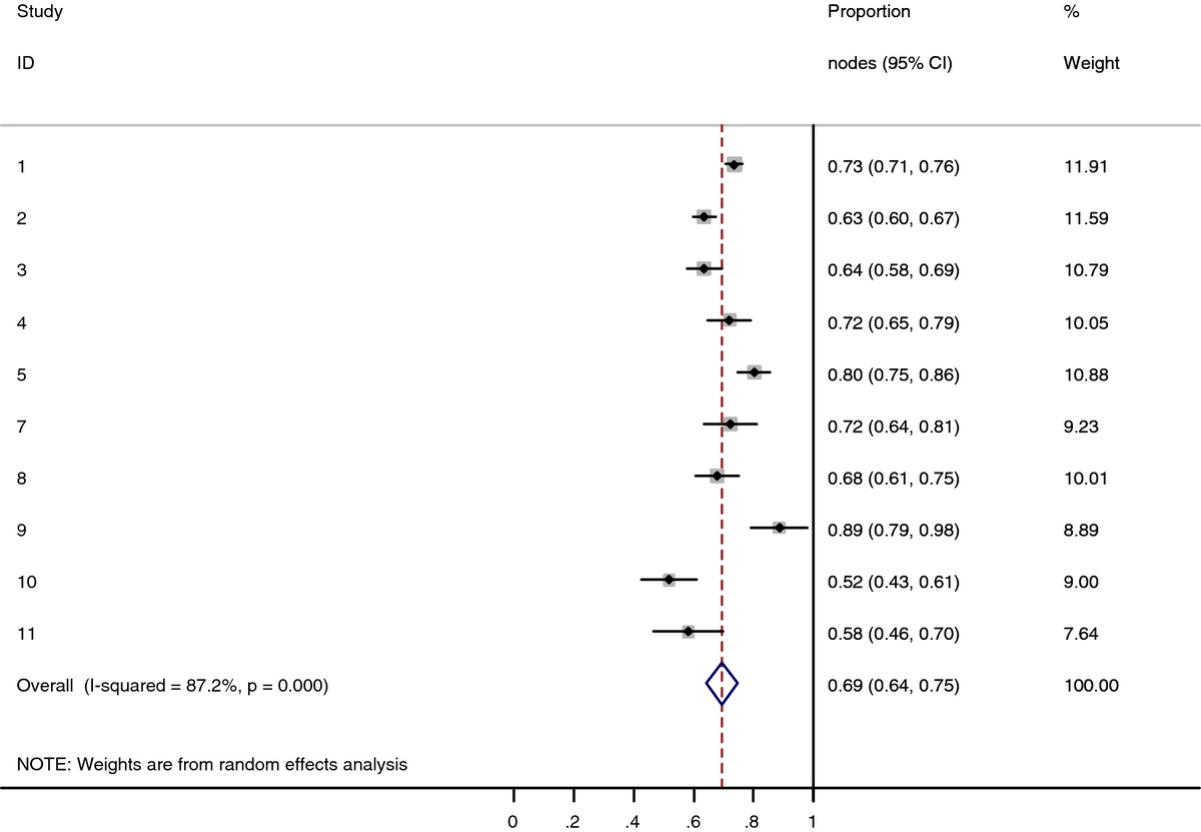
**Meta-analysis plot for patients with RLN metastasis**. This is the forest plot generated from the 10 studies that contained relevant data. The 1^st ^column specifies the study set used. The second column specified the proportion of patients that presented with lymph node metastasis in the particular study. The last column states the weightage of each study contributing to the meta-analysis. The X axis represents the proportion of patients who present with any RLN involvement.

**Figure 6:**
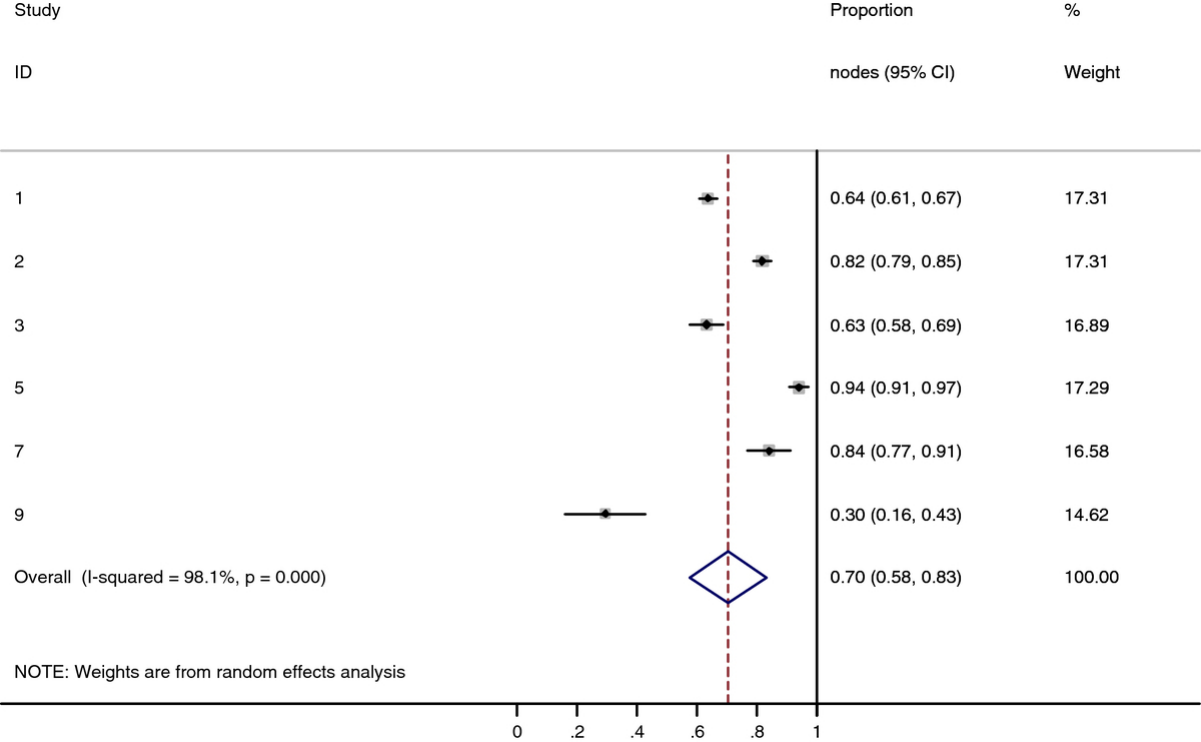
**Meta-analysis plot for patients with Level II cervical LN metastasis**. This is the forest plot generated from the 6 studies that contained relevant data. The 1^st ^column specifies the study set used. The second column specified the proportion of patients that presented with lymph node metastasis in the particular study. The last column states the weightage of each study contributing to the meta-analysis. The X axis represents the proportion of patients who present with any Level II cervical involvement.

**Figure 7:**
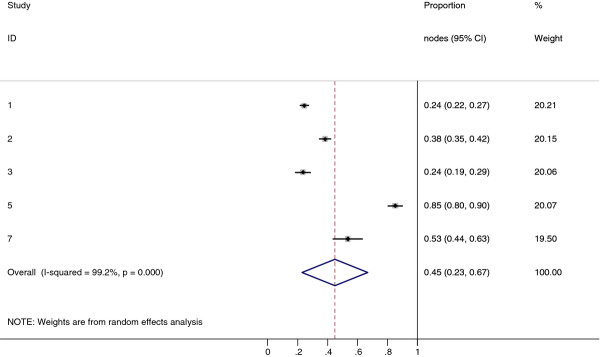
**Meta-analysis plot for patients with Level III cervical LN metastasis**. This is the forest plot generated from the 5 studies that contained relevant data. The 1^st ^column specifies the study set used. The second column specified the proportion of patients that presented with lymph node metastasis in the particular study. The last column states the weightage of each study contributing to the meta-analysis. The X axis represents the proportion of patients who present with any Level III cervical involvement.

**Figure 8:**
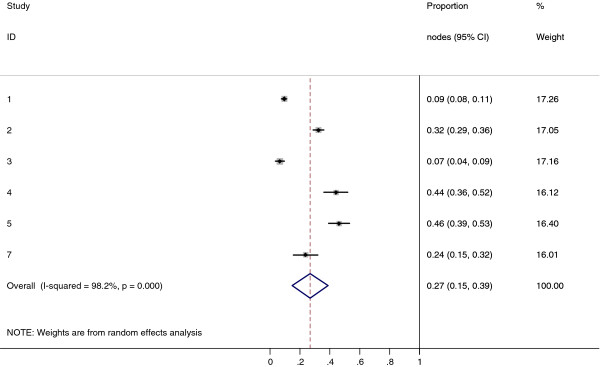
**Meta-analysis plot for patients with Level V cervical LN metastasis**. This is the forest plot generated from the 6 studies that contained relevant data. The 1^st ^column specifies the study set used. The second column specified the proportion of patients that presented with lymph node metastasis in the particular study. The last column states the weightage of each study contributing to the meta-analysis. The X axis represents the proportion of patients who present with any Level V cervical involvement.

**Figure 9:**
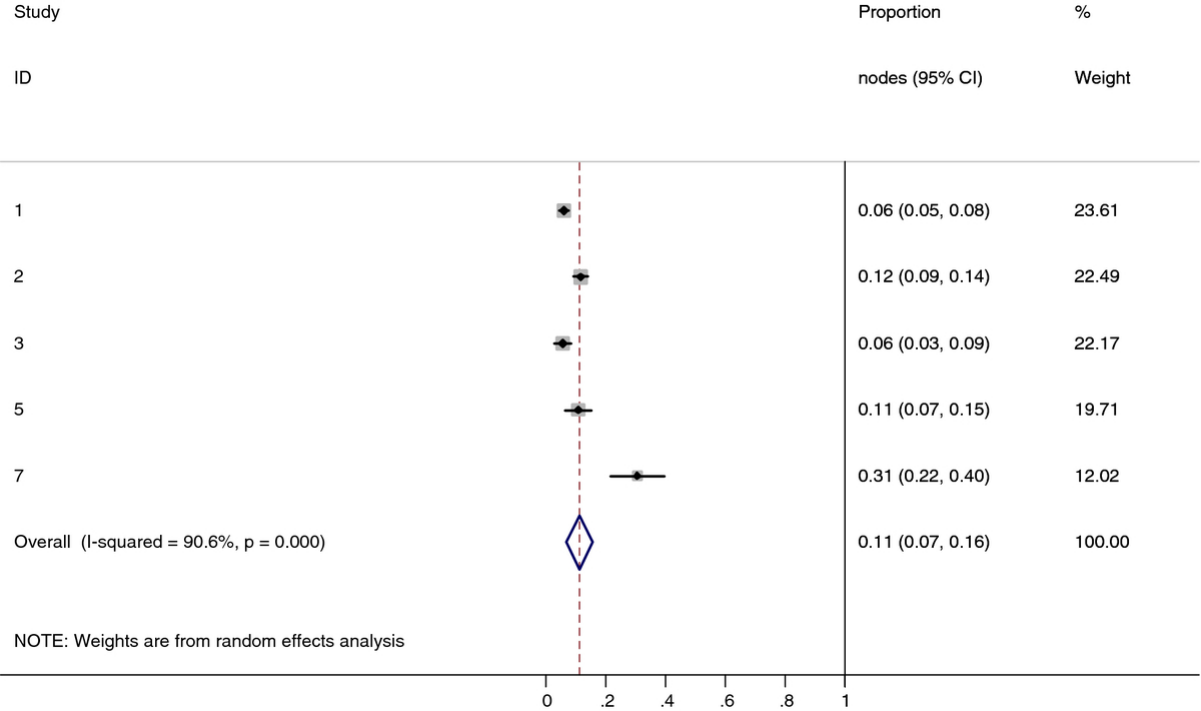
**Meta-analysis plot for patients with Level IV cervical LN metastasis**. This is the forest plot generated from the 5 studies that contained relevant data. The 1^st ^column specifies the study set used. The second column specified the proportion of patients that presented with lymph node metastasis in the particular study. The last column states the weightage of each study contributing to the meta-analysis. The X axis represents the proportion of patients who present with any Level IV cervical involvement.

**Figure 10:**
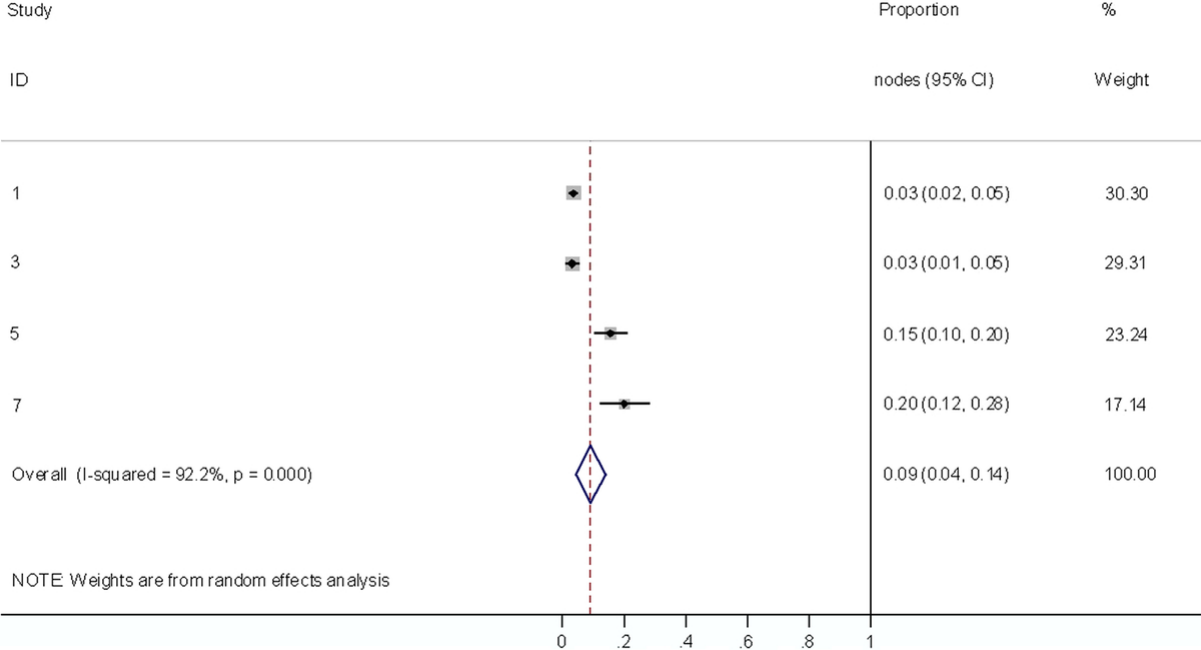
**Meta-analysis plot for patients with SCF LN metastasis**. This is the forest plot generated from the 4 studies that contained relevant data. The 1^st ^column specifies the study set used. The second column specified the proportion of patients that presented with lymph node metastasis in the particular study. The last column states the weightage of each study contributing to the meta-analysis. The X axis represents the proportion of patients who present with any SCF LN involvement.

**Figure 11:**
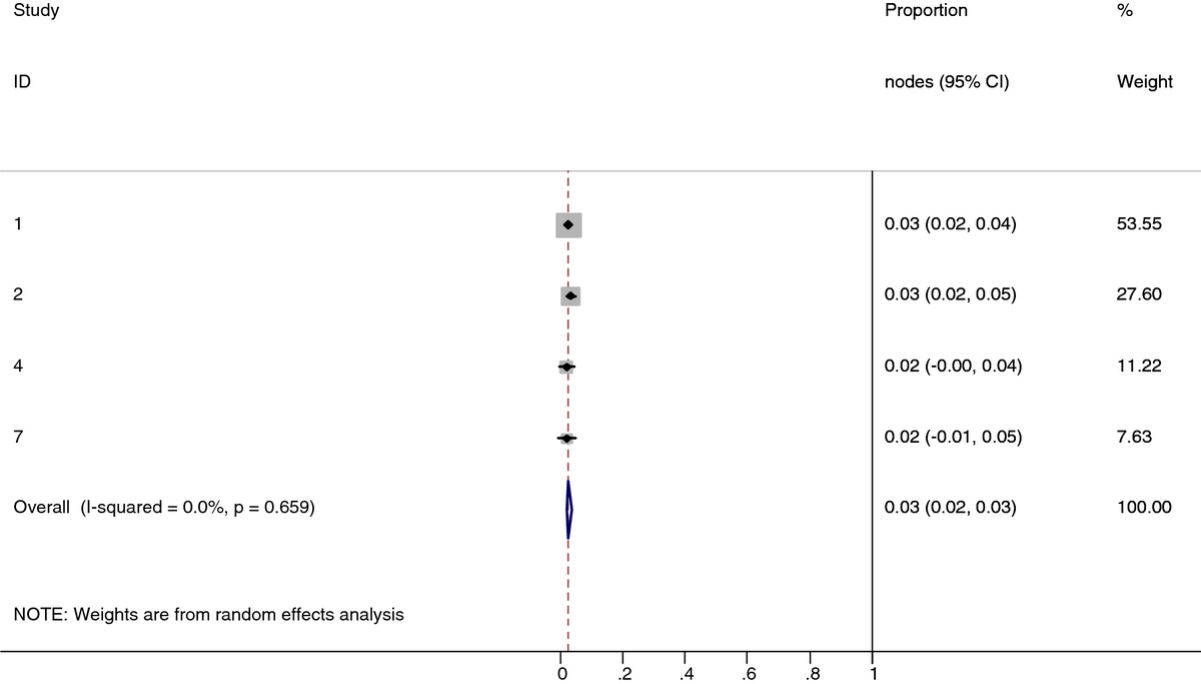
**Meta-analysis plot for patients with Level IB cervical LN metastasis**. This is the forest plot generated from the 4 studies that contained relevant data. The 1^st ^column specifies the study set used. The second column specified the proportion of patients that presented with lymph node metastasis in the particular study. The last column states the weightage of each study contributing to the meta-analysis. The X axis represents the proportion of patients who present with any Level IB cervical involvement.

**Figure 12:**
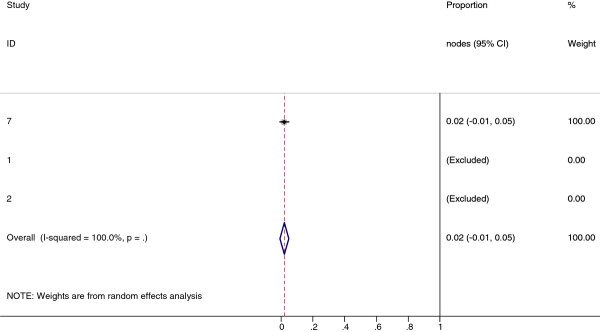
**Meta-analysis plot for patients with Level VI cervical LN metastasis**. This is the forest plot generated from the 3 studies that contained relevant data. The 1^st ^column specifies the study set used. The second column specified the proportion of patients that presented with lymph node metastasis in the particular study. The last column states the weightage of each study contributing to the meta-analysis. The X axis represents the proportion of patients who present with any Level VI cervical involvement.

**Figure 13:**
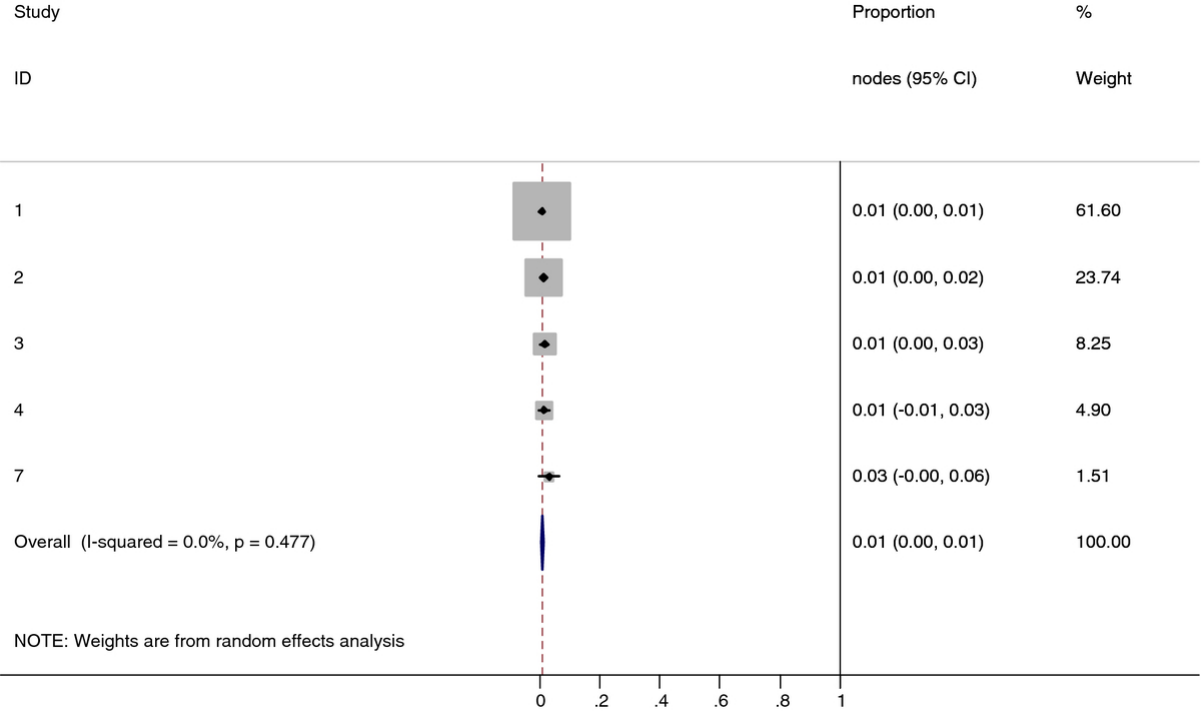
**Meta-analysis plot for patients with parotid LN metastasis**. This is the forest plot generated from the 5 studies that contained relevant data. The 1^st ^column specifies the study set used. The second column specified the proportion of patients that presented with lymph node metastasis in the particular study. The last column states the weightage of each study contributing to the meta-analysis. The X axis represents the proportion of patients who present with any parotid LN involvement.

**Figure 14:**
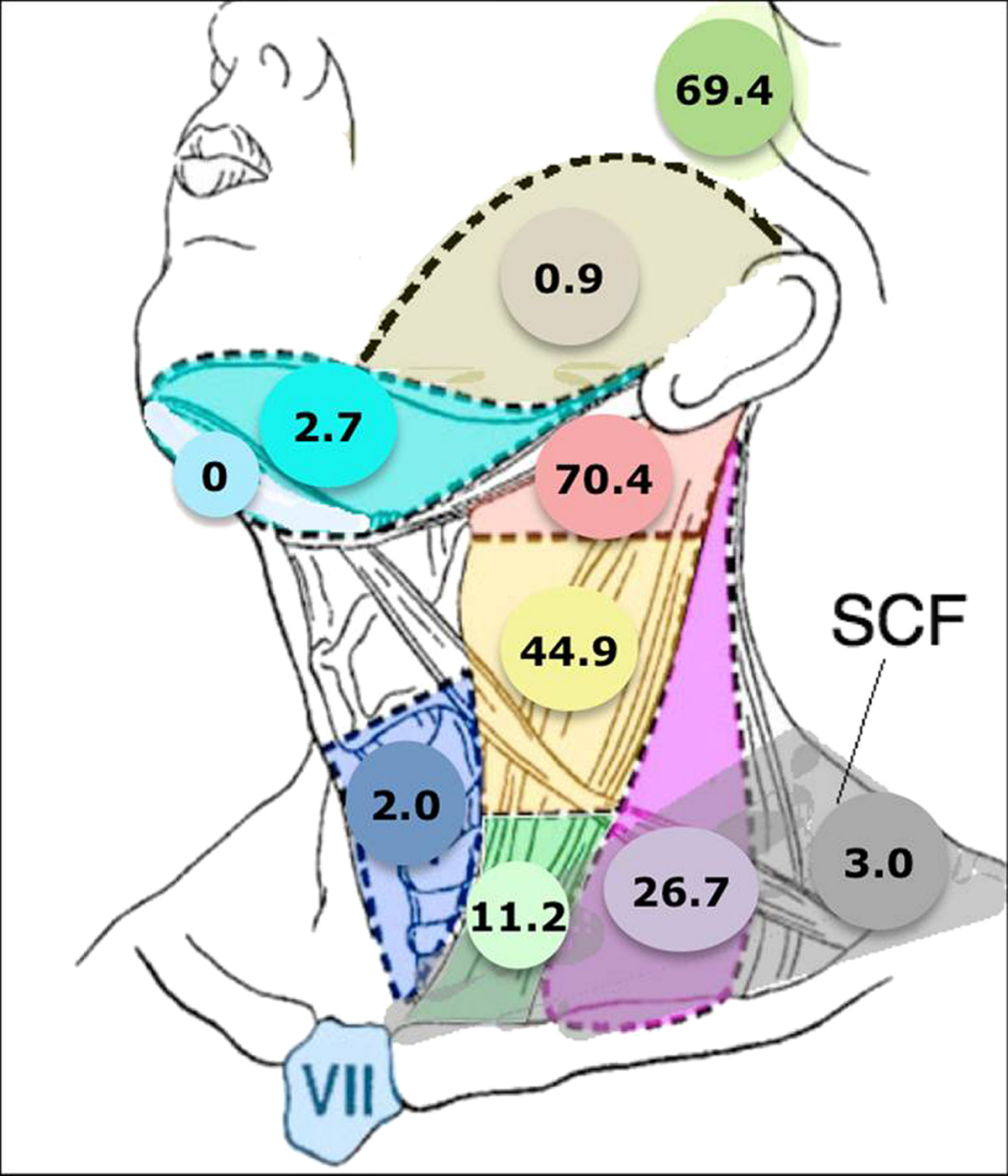
**Pictorial summary of incidence of LN metastasis in NPC**. This is a pictorial representation of the neck. The respective numbers represent the overall percentage of NPC patients presenting with positive LN metastasis at the particular nodal station.

Collectively, 84.9% of NPC cases presented with regional lymphadenopathy. Metastases to neck nodes follow an orderly pattern and the probability of "skip" metastasis between regional nodes vary from 0.5% to 7.9%. Figure 2 summarizes the key findings by nodal levels. Broadly speaking, the nodal stations may be divided into high, intermediate and low risk echelons. The two most commonly involved regions at staging were the RLN (69.4%) and level II LN (70.4%). These stations probably represent the first echelon nodes draining the nasopharynx. Overall probability of levels III, IV, and V nodal involvement are 44.9%, 11.2%, and 26.7%, respectively. These stations are likely to represent the 2^nd ^echelon of draining nodes in NPC. The 3^rd ^echelon of draining nodes in NPC include the supraclavicular, levels IA, IB, and VI nodes, as well as parotid LN, with a rate of involvement at 8.8% (3% if classified according to the consensus guidelines), 0%, 2.7%, 2%, and 0.9% respectively. These likely represent the low risk nodal groups in NPC. Figure 3 and 4 shows meta-analysis plots for lymph node involvement in general and in the cervical region, respectively. Figures 5 and 6 demonstrate meta-analysis plots for the retropharyngeal lymph nodes and level II cervical lymph nodes, which fall into the high risk nodal stations. Figures 7, 8 and 9 demonstrate meta-analysis plots for the level III, level V & level IV cervical lymph nodes, the intermediate risk nodal stations. Figures 10, 11, 12 and 13 demonstrate meta-analysis plots for the supraclavicular, level Ib, level VI & parotid lymph nodes which then fall into the low risk nodal stations. Finally, Figure 14 illustrates the summary incidence rates of nodal metastases for the different levels. There were no patients with positive level Ia cervical lymph nodes, hence the absence of a meta-analysis plot for this group.

## Discussion

### Summary of results

Nasopharyngeal cancer (NPC) has a high propensity of cervical node metastasis. The results of this meta-analysis based on 13 clinical trials using MRI for diagnosis and staging for NPC revealed that the most commonly involved cervical lymph node regions include lateral retropharyngeal nodes and level II nodes with an overall probability of 69.4% & 70.4% respectively for metastasis. These first echelon nodal groups are followed by levels III, VA, and IV, with probabilities of 44.9%, 26.7%, 11.2%, respectively. Certain cervical lymph node groups, including level I, level VI, parotid and supraclavicular nodes have a very low risk for metastasis. An important finding was that lymphatic spread in cervical nodal chain from NPC primary follows an orderly fashion. There was a very low risk of 0.5% in skip nodal metastasis [14].

These findings are important for the management of NPC, particularly in defining proper treatment fields for definitive radiation therapy using conformal technology such as intensity-modulated radiation therapy (IMRT). As the subclinical involvement of cervical lymph nodes cannot be reliably detected by image studies including CT, MRI, and/or PET/CT, proper selection and delineation of clinical target volume for elective irradiation represents a major challenge. Bilateral cervical lymph node metastases usually occur in the early phase of disease development. Therefore, irradiation of the entire cervical lymphatic draining region has been a common practice in radiotherapy of NPC, including stage I disease,[3,28,29] with radiation portals encompassing all levels of cervical lymph nodes from IB to V, including the supraclavicular region [30]. Despite improved outcomes in terms of locoregional control and disease-free survival rates with IMRT,[31,32] such a treatment strategy might represent over-treatment using the current diagnostic and therapeutic technology. NPC patients who are cured of their disease may suffer from the long-term complications from treatment,[5] including xerostomia, neck fibrosis, telangiectasia, thyroid dysfunction, brachial plexopathy and second malignancies, which can significantly impact on function, quality of life or life expectancy. While some of these side effects have been minimized with the advent of conformal radiotherapy, they cannot be fully prevented, especially if the nodal clinical target volumes are adjacent to the critical structures.

An effective strategy in reducing treatment-induced morbidity is to minimize the field for elective radiation in the uninvolved neck region. In a recently reported study by Lin *et al*, exclusion of level Ib lymph nodes and supraclavicular region for elective treatment in IMRT for locoregionally advanced NPC did not reduce the probability of regional control rate as compared to historic controls [33]. Furthermore, in a recently published study of more than 400 NPC patients with N0 disease who were treated with definitive dose of radiation to the primary and upper neck fields (levels II, III, and Va) only, recurrence out of the radiation field at the level IV neck region and supraclavicular area occurred in only one patient [34]. A similar retrospective review of 924 NPC patients with N0 disease compared the inferior border of radiotherapy either at the cricoid cartilage or below the cricoid cartilage revealed no statistically significant difference between the two groups [14]. The use of MRI in the diagnosis and staging and/or more advanced treatment strategies including IMRT and concurrent chemoradiation therapy might play substantial roles for the aforementioned findings. However, the optimal strategy of selection and delineation of the sub clinical regional disease in clinical target volume (CTV) in the treatment of NPC has not been well addressed. Knowledge on regional lymph node drainage in NPC diagnosed and staged in the modern era particular with MRI is limited, and the current available data are usually not complete with inconsistent results. More systemic and comprehensive understanding of the patterns of cervical nodal involvement in NPC is clearly necessary for proper design of clinical trials using conformal radiation techniques and will provide practice-changing clinical evidence.

Although our results represented the most comprehensive and conclusive data for the pattern and probability of cervical lymph node spread in NPC, a number of issues related to the design and analyses need to be addressed. As radiation therapy is the only curative treatment currently, and surgery including neck dissection has a limited role in the primary treatment for NPC, adenopathy is universally diagnosed by imaging studies. Histological diagnosis for cervical node metastasis is rarely performed. The radiology diagnosis of cervical lymph adenopathy is largely based on size and morphology criteria [35] derived from surgical series. Prior to the use of MRI for staging and diagnosis, NPC was usually evaluated using contrast enhanced CT to assess the extent of disease in both primary and neck regions [36]. Most of the available data on neck node involvement and its treatment are based on CT imaging. However, the sensitivity and specificity rates of enhanced CT for the diagnosis of cervical lymph adenopathy are approximately 14-60% and 78-92% respectively, compared to 29-80% and 82-92% respectively for MRI [35]. The benefit of MRI over CT in evaluating cervical lymph nodes for NPC has also been recently reported [37]. Additionally, a meta-analysis has shown that the accuracy of MRI appears superior to PET-CT in the evaluation of cervical lymphadenopathy [38]. Accordingly, the updated 7^th ^edition of the TNM Classification of Malignant Tumours [39] proposes that MRI should be considered the standard imaging modality for the diagnosis and staging of NPC.

In addition, a locally advanced tumor in the nasopharynx obviously would have a higher probability of cervical lymph node metastasis as compared to early stage disease. The knowledge of the exact NPC stage of each study subject could provide valuable insight as to how nodal metastasis changes with staging of the tumor. Unfortunately, this information was not readily available for such analyses.

One of the greatest challenges in performing this meta-analysis was the weighting of the individual studies. The studies were highly heterogeneous in their study design from prospective to retrospective (predominant). Many of them were not designed to study regional nodal metastasis as the main end point but were included due to the paucity of such data in the MRI era. As such, the usual criteria for weighting studies in systematic reviews could not be applied to this study [40,41].

## Conclusion

This meta-analysis provides some grounds to potentially reduce treatment volume in NPC patients diagnosed and staged using MRI and treated with modern radiotherapy technology such as IMRT. However, clinical studies are required before this volume reduction can be adopted as standard of care. According to our results, we hypothesize that limiting coverage to the retropharyngeal, levels II, III, and Va nodes in patients with N0 disease or on the uninvolved neck in patients with N1 disease would not compromise regional control rates and disease-free survival. Furthermore, the rarity of skip metastasis in NPC lymphatic drainage provides a basis to eliminate irradiation to the entire involved side of the neck, and only encompass the echelon inferior to the involved level. This hypothesis will be tested in a newly designed multi-center prospective clinical trial initiated at our institute.

## Abbreviations

NPC: nasopharyngeal carcinoma; LN: lymph node; No: number; SCF: supraclavicular fossa; RLN: retropharyngeal lymph node.

## Competing interests

The authors declare that they have no competing interests.

## Authors' contributions

FH and JL conceived the study. Data was acquired independently by JL and FH. AE and IT undertook data analysis and interpretation. FH and LKM prepared the manuscript with contributions from all co-authors. All authors read and approved the final manuscript

## Pre-publication history

The pre-publication history for this paper can be accessed here:

http://www.biomedcentral.com/1471-2407/12/98/prepub
